# Draft genome sequence of *Rosettibacter* sp. KY25 isolated from a terrestrial hot spring, Japan

**DOI:** 10.1128/mra.01464-25

**Published:** 2026-03-17

**Authors:** Shigeru Kawai, Yuya Tsukamoto, Yusuke Tsukatani, Yuu Hirose, Arisa Nishihara

**Affiliations:** 1 Department of Applied chemistry and Life Science, Toyohashi University of Technology 13129, Aichi, Japan; 2 Department of Earth Science, Tohoku University 13101, Miyagi, Japan; 3 Japan Agency for Marine-Earth Science and Technology (JAMSTEC), Biogeochemistry Research Center (BGC), Kanagawa, Japan; 4 Department of Life Science and Biotechnology, The National Institute of Advanced Industrial Science and Technology, Ibaraki, Japan; University of Wisconsin-Madison, Madison, Wisconsin, USA

**Keywords:** *Rosettibacter*, hot spring, genome analysis

## Abstract

We report here the draft genome sequence of a thermophilic heterotrophic bacterium, *Rosettibacter* sp. strain KY25, isolated from hot spring-associated microbial mats in Japan. The genome of KY25 is 3,177,379 bp in length and contains 2,668 predicted coding sequences, which include genes required for aerobic respiration, nitrate reduction, and sugar utilization.

## ANNOUNCEMENT

*Rosettibacter* is a genus of thermophilic, chemoheterotrophic bacteria belonging to the phylum *Ignavibacterota* (1). The type strain of the genus *Ignavibacterium* was isolated from microbial mats at a terrestrial hot spring and was initially regarded as a non-photosynthetic member of the *Chlorobi* (green sulfur bacteria). Subsequent phylogenomic analyses reclassified it within the phylum *Ignavibacterota* (2). To date, the phylum comprises two families, five genera, and six species, all of which are thermophilic (1–5). Isolates of the genus *Rosettibacter* have been obtained only from Russian hot springs, and its geographic and ecological range therefore remains poorly understood. Here, we report the draft genome sequence of a *Rosettibacter* sp. strain isolated from a Japanese hot spring. Hot spring-associated microbial mats were collected from Kawara-no-Yukko spring (38°57′37.9″N 140°31′45.3″E) at 56°C using sterile tweezers and conical tubes. A piece of the mat was suspended in the hot-spring water, spread onto 1/5 PE medium agar plates (PE medium, diluted to one-fifth the concentration of organic components [6]), and cultivated at 50°C under aerobic conditions in the dark to minimize lid condensation. The single orange-colored colony was subcultured three times on agar plates, yielding strain KY25. The 16S rRNA gene was amplified using universal primers 27F/1492R, and the sequence showed 99.0% and 97.4% identity with *R. primus* JCM 39,244 (JAYKGK010000006.1) and *R. firmus* JCM 39,243 (JAYKGJ010000002.1), respectively.

Genomic DNA was extracted from cells of strain KY25 using the DNeasy UltraClean Microbial Kit (QIAGEN). The sequencing library was prepared using the MGIEasy Circularization Kit and DNBSEQ-G400RS High-throughput Sequencing Kit (MGI Tech). The library was sequenced on the DNBSEQ-G400 platform, generating 2 × 150 bp paired-end reads. Raw reads (13,999,216 paired-end reads) were adapter-trimmed and quality-controlled using Trimmomatic v.0.39 (7) and quality-checked using FastQC v.0.12.1 (8). Genome assembly was performed using SPAdes v.3.14.0 (9). After assembly, contigs longer than 1000 bp were retained for downstream analyses. Genome annotation was performed with Prokka v.1.14.6 (10). Unless otherwise noted, default parameters were used for all software.

The draft genome of strain KY25 is 3,177,379 bp with an N50 of 141,312 bp, with 100% of reads mapping and an estimated 660× coverage. The genome completeness is estimated to be 99.89% using CheckM2 (11). G+C content of the genome is 31%. Genome annotation software predicted 2,668 protein-coding sequences, 3 rRNAs, and 44 tRNAs from 58 contigs. The average nucleotide identity (ANI) values showed 84.75% and 78.63% against *R. primus* JCM39244 (JAYKGK010000006.1) and *R. firmus* JCM39243 (JAYKGJ010000002.1), respectively. The genome lacks photosynthesis-related and Calvin-cycle-related genes but encodes complete respiratory and denitrifying electron-transport chains, suggesting versatile chemoheterotrophic metabolism capable of oxygen-dependent respiration in the presence of O_2_ and nitrate- and nitrite-dependent respiration in the absence of O_2_, similar to that observed in some members of the *Melioribacteraceae*. The presence of genes for numerous ABC transporters that facilitate the uptake of peptides, sugars, and metals suggests a broad substrate capacity and environmental adaptability.

## Data Availability

This whole-genome shotgun project has been deposited at DDBJ/ENA/GenBank under the accession number BAAJAO010000000. The raw sequence reads are available in the DDBJ Sequence Read Archive (DRA) under database accession number DRR805793 and BioProject accession number PRJDB38097.

## References

[1] Podosokorskaya OA, Petrova NF, Tikhonova EN, Klyukina AA, Elcheninov AG. 2024. Rosettibacter primus gen. nov., sp. nov., and Rosettibacter firmus sp. nov., facultatively anaerobic moderately thermophilic bacteria of the class Ignavibacteria from hot springs of North Ossetia. Syst Appl Microbiol 47:126528. doi:10.1016/j.syapm.2024.126528 38959749

[2] Podosokorskaya OA, Kadnikov VV, Gavrilov SN, Mardanov AV, Merkel AY, Karnachuk OV, Ravin NV, Bonch-Osmolovskaya EA, Kublanov IV. 2013. Characterization of Melioribacter roseus gen. nov., sp. nov., a novel facultatively anaerobic thermophilic cellulolytic bacterium from the class Ignavibacteria, and a proposal of a novel bacterial phylum Ignavibacteriae. Environ Microbiol 15:1759–1771. doi:10.1111/1462-2920.12067 23297868

[3] Iino T, Mori K, Uchino Y, Nakagawa T, Harayama S, Suzuki KI. 2010. Ignavibacterium album gen. nov., sp. nov., a moderately thermophilic anaerobic bacterium isolated from microbial mats at a terrestrial hot spring and proposal of Ignavibacteria classis nov., for a novel lineage at the periphery of green sulfur bacteria. Int J Syst Evol Microbiol 60:1376–1382. doi:10.1099/ijs.0.012484-0 19671715

[4] Podosokorskaya OA, Merkel AY, Novikov AA, Klyukina AA, Elcheninov AG. 2025. Pyranulibacter aquaticus gen. nov., sp. nov., the first nitrate-reducing representative of the class Ignavibacteria from an Iturup well. Syst Appl Microbiol 48:126639. doi:10.1016/j.syapm.2025.126639 40664022

[5] Podosokorskaya OA, Elcheninov AG, Gavrilov SN, Petrova NF, Klyukina AA, Zavarzina DG, Merkel AY. 2023. New representatives of the class Ignavibacteria inhabiting subsurface aquifers of Yessentuki mineral water deposit. Water (Basel) 15:3451. doi:10.3390/w15193451

[6] Hanada S, Hiraishi A, Shimada K, Matsuura K. 1995. Chloroflexus aggregans sp. nov., a filamentous phototrophic bacterium which forms dense cell aggregates by active gliding movement. Int J Syst Bacteriol 45:676–681. doi:10.1099/00207713-45-4-676 7547286

[7] Bolger AM, Lohse M, Usadel B. 2014. Trimmomatic: a flexible trimmer for Illumina sequence data. Bioinformatics 30:2114–2120. doi:10.1093/bioinformatics/btu170 24695404 PMC4103590

[8] Andrews S. 2010. FastQC: a quality control tool for high throughput sequence data. Available from: https://www.bioinformatics.babraham.ac.uk/projects/fastqc/

[9] Bankevich A, Nurk S, Antipov D, Gurevich AA, Dvorkin M, Kulikov AS, Lesin VM, Nikolenko SI, Pham S, Prjibelski AD, Pyshkin AV, Sirotkin AV, Vyahhi N, Tesler G, Alekseyev MA, Pevzner PA. 2012. SPAdes: a new genome assembly algorithm and its applications to single-cell sequencing. J Comput Biol 19:455–477. doi:10.1089/cmb.2012.0021 22506599 PMC3342519

[10] Seemann T. 2014. Prokka: rapid prokaryotic genome annotation. Bioinformatics 30:2068–2069. doi:10.1093/bioinformatics/btu153 24642063

[11] Chklovski A, Parks DH, Woodcroft BJ, Tyson GW. 2023. CheckM2: a rapid, scalable and accurate tool for assessing microbial genome quality using machine learning. Nat Methods 20:1203–1212. doi:10.1038/s41592-023-01940-w 37500759

